# The Diabetic Foot

**DOI:** 10.1155/2017/3585617

**Published:** 2017-10-10

**Authors:** I. Migdalis, L. Czupryniak, N. Lalic, R. D. Leslie, N. Papanas, P. Valensi

**Affiliations:** ^1^Second Medical Department and Diabetes Centre, NIMTS Hospital, 12 Monis Petraki, 11521 Athens, Greece; ^2^Department of Diabetology and Internal Medicine, Warsaw Medical University, Warsaw, Poland; ^3^Clinic for Endocrinology, Diabetes and Metabolic Diseases, CCS, Faculty of Medicine, University of Belgrade, Belgrade, Serbia; ^4^Department of Diabetes, Saint Bartholomew's Hospital, University of London and Blizard Institute, EC1A 7BE, London, UK; ^5^Diabetic Foot Clinic-Diabetes Centre, Second Department of Internal Medicine, Democritus University of Thrace, 68100 Alexandroupolis, Greece; ^6^Department of Endocrinology, Diabetology and Nutrition, Jean Verdier Hospital, AP-HP, Paris Nord University, CRNH-IdF, CINFO, 93140 Bondy, France

Diabetic foot ulcers remain a serious medical problem, which is extremely difficult to heal and exhibits a high recurrence rate [1]. Thus, it is continuously receiving increased scientific attention, in an effort to improve outcomes [2–4]. There is ongoing progress in peripheral arterial disease [1], neuropathy [1, 5], off-loading [1, 2], infection [1, 2], and wound healing [1, 2]. The present special issue is devoted to new research in the field of diabetic foot.

S. Yang et al. in their paper entitled “Alcohol Consumption Is a Risk Factor for Lower Extremity Arterial Disease in Chinese Patients with T2DM” reported that alcohol consumption was a significant independent risk factor for peripheral arterial disease in hospitalized Chinese patients with type 2 diabetes, and this finding has obvious practical implications.

In their experimental paper “Investigation of the Effects and Mechanisms of Mai Tong Formula on Lower Limb Macroangiopathy in a Spontaneous Diabetic Rat Model,” G. Gong et al. examined a new Chinese herbal formula, which has recently been used to treat peripheral arterial disease in diabetes. In the spontaneous diabetic rat model, they found that this formula reduced fasting blood glucose, triglycerides, total cholesterol, interleukin-6, and vascular endothelial growth factor, while it increased serum insulin. Histology and ultrasonography provided evidence that treatment also reduced endothelial dysfunction and injury. More experience is anticipated.

T. Didangelos et al. in their article “Efficacy of Administration of an Angiotensin Converting Enzyme Inhibitor for Two Years on Autonomic and Peripheral Neuropathy in Patients with Diabetes Mellitus” examined the effect of a 2-year quinapril treatment on diabetic cardiovascular autonomic neuropathy and peripheral neuropathy. They documented improvement in cardiovascular autonomic neuropathy, mainly parameters of parasympathetic dysfunction, and this may merit further clinical utilization.

Z. Zheng et al. in their article “Sympathetic Denervation Accelerates Wound Contraction but Inhibits Reepithelialization and Pericyte Proliferation in Diabetic Mice” turned their attention to the impact of sympathetic denervation using intraperitoneal 6-hydroxydopamine administration on inflammation, angiogenesis, and wound healing in diabetic mice. They found that treatment decreased epidermal growth factor, hindering reepithelization, and it impaired pericyte proliferation. However, it enhanced wound contraction by reducing interleukin-1*β* and mast cells.

A. Watanabe et al. in their paper entitled “Development of a Plantar Load Estimation Algorithm for Evaluation of Forefoot Load of Diabetic Patients during Daily Walks Using a Foot Motion Sensor” used a motion sensor attached to each shoe, in order to obtain forefoot acceleration and angular velocity data. This promising modality identified excessive forefoot loading during 3-hour daily walks of 2 diabetic patients. It may now be further employed to ascertain differences between walking on level ground and on slopes/stairs.

J. Zhao et al. in their manuscript entitled “Therapeutic Effects of Static Magnetic Field on Wound Healing in Diabetic Rats” looked at the effects of static magnetic field on incisional wound healing in streptozotocin-induced diabetic rats. In comparison with sham magnetic treatment, static magnetic field accelerated healing and increased tissue strength. More experience in humans would be welcome.

X. Zhang et al. in their paper “NLRP3 Inflammasome Expression and Signaling in Human Diabetic Wounds and in High Glucose Induced Macrophages” included patients with type 2 diabetes and chronic foot wounds. They demonstrated higher expression of the NLRP3 inflammasome, caspase-1, and interleukin-1*β* in comparison with wounds in nondiabetic patients. The higher expression was confirmed at both mRNA and protein level. These findings should be interpreted in the context of increased inflammation in diabetic foot ulcerations.

Finally, M. Al-Hariri in the review article entitled “Sweet Bones: The Pathogenesis of Bone Alteration in Diabetes” outlined bone changes in diabetes. These mainly relate to deficits in mineralization, decreased osteoid surface, osteoblast activity, accumulation of advanced glycation end products, and oxidative stress, as well as increased urinary excretion of calcium and magnesium. Certainly, bone pathology in diabetes deserves further attention, but we should also take into consideration the effects of some antidiabetic agents [6].

There are considerable new research data in miscellaneous issues pertaining to the diabetic foot. New investigations need to be carefully interpreted, so that they can mature into useful clinical implications. This process is vital for improved management of the diabetic foot [1, 3].

*I. Migdalis* *L. Czupryniak* *N. Lalic* *R. D. Leslie* *N. Papanas* *P. Valensi*
